# Controlling the laxative abuse of anorexia nervosa patients with the Serigaya Methamphetamine Relapse Prevention Program workbook: a case report

**DOI:** 10.1186/s13030-019-0166-z

**Published:** 2019-10-23

**Authors:** Kuniyoshi Toyoshima, Ichiro Kusumi

**Affiliations:** 1 Department of Psychiatry, Wakkanai City Hospital, Wakkanai, Japan; 2 Department of Psychiatry, Graduate School of Medicine, Hokkaido University, Kita 15, Nishi 7, Sapporo, 060-8638 Japan

**Keywords:** Addiction, Anorexia nervosa-binge eating/purging type, Laxative abuse, Matrix model, SMARPP workbook

## Abstract

**Background:**

There is no consensus on effective treatment for laxative abuse in patients with eating disorders. Here, we report the case of a patient with laxative abuse who showed some improvement through an intervention based on the Matrix model.

**Case presentation:**

A woman diagnosed with anorexia nervosa-binge eating/purging type (AN-BP) steadfastly denied laxative abuse and would not admit to suffering from an eating disorder. This led to low motivation for undergoing conventional psychotherapy, psychoeducation, and cognitive behavioral therapy. These were ineffective and followed by repeated cycles of hospitalization and discharge. The patient’s general condition, as depicted by her laboratory and clinical parameters, deteriorated due to the medical complications resulting from laxative abuse.

Focusing on laxative abuse, we considered an intervention for drug addiction. Because the patient could maintain a diet diary and acknowledged laxative abuse as a drug addiction, we introduced the Serigaya Methamphetamine Relapse Prevention Program (SMARPP) workbook as a self-administered treatment. The patient meticulously completed the treatment and experienced a gradual improvement in laxative abuse. She has not been re-hospitalized in 4 years, currently performs household chores, and demonstrates improved social function.

**Conclusions:**

In patients with AN-BP, the SMARPP workbook may be effective in treating laxative abuse.

## Background

Anorexia Nervosa (AN) constitutes a debilitating and often fatal eating disorder, wherein the restraint of appetite and emotion occurs simultaneously with an obsessive-compulsive behavior in addition to a cognitive disposition that demonstrates significant attention to detail [1]. The compulsivity of AN has sometimes raised the notion that the condition may embed some degree of addictive behavior [2]. In AN patients with low body weight, their physiological systems are affected, resulting in conditions ranging from hypotension and osteopenia to life-threatening arrhythmias. The electrolyte anomalies that occur in patients with purging or frequent laxative abuse often necessitates rapid treatment [3]. Reportedly, family-based treatment is effective for adolescent patients with AN [4]. This may be due to the shorter duration of illness that adolescent patients experience in comparison to adult patients as well as their tendency to live around family members who can provide moral and emotional support that may disincentivize their use of laxatives. However, the efficacy of this approach remains unclear for middle-aged patients [5].

Recent evidence-based therapeutic advances for adult patients with AN include cognitive remediation therapy, exposure therapy, and non-invasive neuromodulation [6]. Although psychotherapy, in addition to cognitive behavioral therapy, is the fundamental treatment for AN, it has not been proven effective in several cases. In fact, patients with purging AN who exhibit both vomiting and laxative abuse manifested the poorest course [7]. This is part of the underlying reason for the proposal that purging behavior should be comprehensively evaluated in patients with eating disorders because it is usually not self-reported due to the associated shame [8].

Against this background, this paper reports the case of a woman with anorexia nervosa-binge eating/purging type (AN-BP), whose state of physical crisis deteriorated due to laxative abuse and led to her recurrent hospitalization. Adopting a drug addiction perspective, we attempted an intervention for laxative abuse using the Serigaya Methamphetamine Relapse Prevention Program (SMARPP) workbook. This is a form of cognitive behavioral therapy that correlates the features of an eating disorder with drug cravings and harm to both the brain and body. On this basis, we evaluated the efficacy of the SMARPP workbook for the aforementioned patient.

## Case presentation

A 49-year-old woman with anorexia nervosa-binge eating/purging type (AN-BP) and a simultaneous history of laxative abuse was admitted to our hospital for the second time. According to her medical history, she had initiated dieting after entering vocational school at the age of 18. Initially, she practiced only dietary restrictions but gradually began self-induced vomiting. Based on the medical history she provided, her weight (50 kg) decreased gradually and was 40 kg by the time she got married at 28. During this time, she had begun consuming a commercially available laxative. By age 35, her body weight was 35 kg. She was diagnosed with impaired renal function at 41 and renal failure occurred at 45; subsequently, hemodialysis was initiated. At this time, she was diagnosed with AN on a visit to a nearby mental clinic, but refused hospitalization.

Throughout this period, the patient continued to restrict her diet and abuse laxatives. At the age of 46, her body weight decreased to < 30 kg, and dialysis became difficult because of the decrease in her blood pressure. She was advised to undergo hospitalization at a specialized medical institution, and in October of the same year, she was admitted to our facility. Her weight was 26.4 kg [body mass index (BMI), 11.9 kg/m^2^], and we confirmed her fear of obesity, body image distortion, and purging behavior, which included self-induced vomiting and laxative abuse. No questionnaire or semi-structured interview was employed to confirm an eating disorder. Rather, the patient was diagnosed with AN-BP using the fourth edition of the Diagnostic and Statistical Manual of Psychiatric Disorders (DSM-IV-TR) [9]. At the onset of her eating disorder, the patient exhibited binge eating and had begun vomiting via laxative use to compensate for her overconsumption.

### Clinical assessments

Table 1 presents the values obtained via our clinical assessments. Cognitive behavioral therapy was administered to the patient using a column technique and nutritional therapy. Her weight increased to 38 kg in May at the age of 47, and she was discharged in August. As soon as she was discharged, she relapsed and resumed laxative abuse. The patient visited our hospital once a week, but her body weight gradually decreased to the 20-kg range. She was re-hospitalized in February at the age of 49. Upon admission, her weight was 33.7 kg (BMI, 15.2 kg/m^2^). Based on the following laboratory indices, serum creatinine (4.43 mg/dl ↑), serum sodium (Na 137 mEq/l ↓), serum potassium (3.0 mEq/l ↓) serum chlorine (102 mEq/l), and blood urea nitrogen (22 mg/dl); an internal medicine specialist made a diagnosis of pre-renal renal failure due to laxative abuse. This led to her placement under maintenance hemodialysis. In addition to systemic management using central venous nutrition, behavioral therapy was also administered, and her body weight gradually increased (+ 5.3 kg/3 months). By May, her body weight was 39 kg (BMI, 17.6 kg/m^2^) after dialysis. The patient was eating a 1600 KCal meal at this time; however, she expressed fear of obesity, body image distortion, and a desire to remain slim. Also, while we found no fecal storage in the X-ray performed each week, she insisted on using a laxative. As such, we surmised that while her original intent for abusing laxatives was to reduce weight, her intention had evolved such that her use of laxatives had become a top priority to eliminate anxiety. This prompted us to adopt a drug addiction intervention.

**Table 1:** Clinical Assessments

	Before SMARPP (May, at the age of 49)	After SMARPP (December, at the age of 49)	Follow-up (January, at 53 years)
GAF scale	35	50	65
Body weight	39 kg	45.5 kg	41.0 kg
BMI	17.6 kg/m^2^	20.5 kg/m^2^	18.5 kg/m^2^
SCOFF-Sick	no	no	no
SCOFF-Control	yes	no	no
SCOFF-One-stone (14 lbs./6.5 kg)	no	no	no
SCOFF-Fat	yes	no	no
SCOFF-Food	yes	no	no

*Abbreviations*: *GAF* global assessment of functioning, *BMI* body mass index, *SCOFF* sick, control, one stone, fat, and food

### Clinical management via SMARPP workbook

We introduced the SMARPP workbook and used it to conceptualize laxative abuse as an addiction. We chose this approach because at the heart of the phenomenon of addiction is the erosion of the desire to refrain from harmful behavior, which is caused by neuroplastic changes in the brain that limit the rational control of such harmful behavior [10]. A clinical examination of the attitude of a patient with AN toward laxative abuse in light of DSM supported our approach. We think this approach is preferable to the use of CBT, which conceptualizes the laxative abuse of AN as only a purging behavior.

SMARPP is based on cognitive behavioral therapy and includes topics on the correlation of an eating disorder with drug cravings and harm to the brain and body. It comprises weekly group sessions, including motivational interviewing attitudes [11] and cognitive behavioral therapy for relapse prevention, following the Matrix model [12]. The SMARPP workbook utilizes the SMARPP principles. The Japanese version was published in 2011 as the “Recovery Support Workbook from Drug–Alcohol Addiction.” It comprises 28 chapters, which both patients and psychiatrists can read and complete together while adjusting to the patients’ pace. Each chapter required approximately 30 min for completion and the patient read the chapter for the first 15 min and answered questions during the next 15 min. Upon completion of each chapter, we evaluated the patient’s impressions. The whole exercise was performed once a week for 7 months in our hospital environment.

**Fig. 1:**
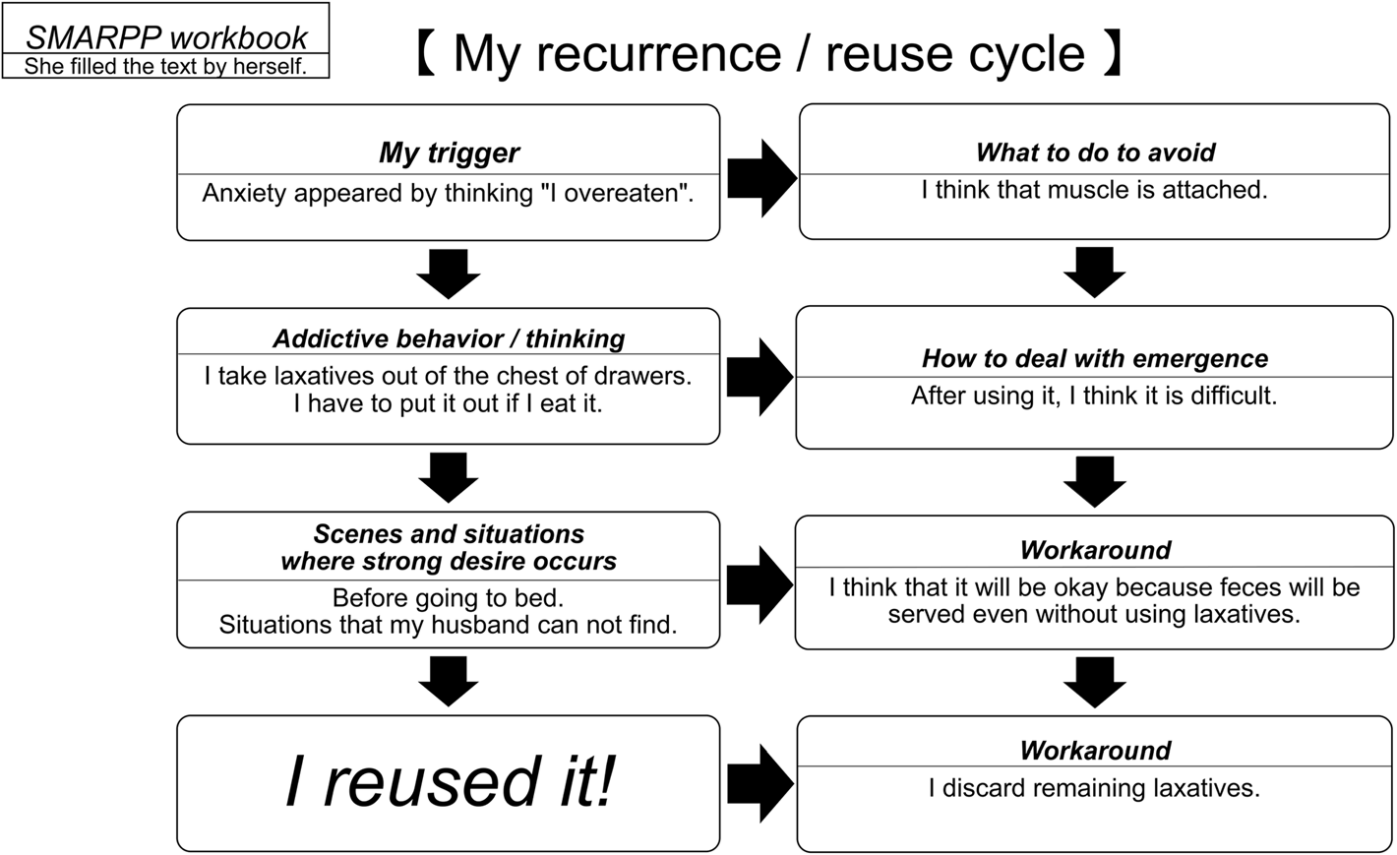
SMARPP workbook. (The patient reviewed the cycle of recurrence, reuse, and ways to manage her disease)

**Fig. 2:**
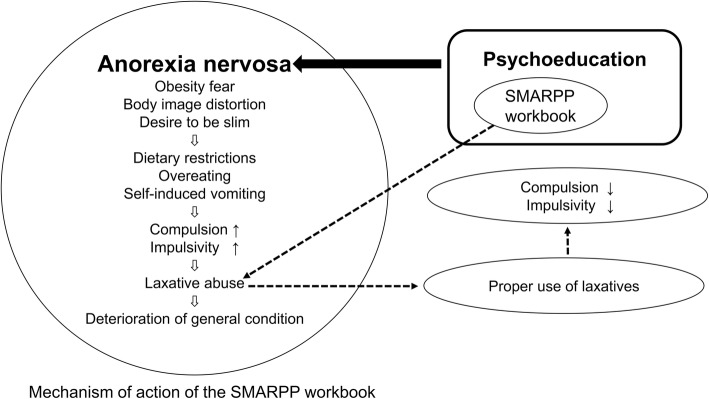
Mechanism of action of the SMARPP workbook

During this period, the patient herself recognized her laxative abuse and admitted to having previously used approximately 30 tablets each night; she also confessed to using approximately 200 tablets. In fact, from August (at the age of 47) to February (at the age of 49), her laxative abuse gradually increased from 30 tablets to 200 tablets per day. The laxatives she used were commercially available (Bisacodyl; 5 mg, and Sennoside A・B; 5.27 mg, per tablet). In December, at the age of 49, she completed the table on the recurrence and reuse cycle in the last session (Fig. 1). In addition, at the conclusion of the SMARPP workbook, she stated, “I thought there was so much damage to my brain and my body.” Nonetheless, her weight continued to increase further, and she was discharged in December at the age of 49 years, once her body weight reached 45.5 kg (BMI, 20.5 kg/m^2^) after dialysis. We examined the effectiveness of the SMARPP workbook as shown in Fig. 2. The SMARPP workbook intervened in the laxative abuse, which resulted in appropriate laxative use and reduced compulsive and impulsive behaviors.

In the cognitive functional battery conducted upon her discharge, the results of the Continuous Performance Test errors improved compared with that performed before her discharge. The Continuous Performance Test (CPT) was applied using a stimulus presentation software (A-X CPT) and lasted for 7 min. This test assesses the participant’s sustained attention and reaction time [13]. Post-discharge, she continued outpatient visits until March, when she reached the age of 53; she has not been re-hospitalized since. The prescription content of her laxative was Sennoside A・B, 48 mg; Sennoside A, B, 2 g; Lubiprostone, 48 μg; and Sodium Picosulfate Hydrate, 15 mg per day, whereas at the time of laxative abuse, it was Bisacodyl, 1000 mg and Sennoside A・B, 1054 mg per day.

From Table 2, it can be seen that overall there were improvements in the blood pressure (from hypotension to normotension) and BMI of our patient with reference to her initial hospitalization, second hospitalization, and her conditions at discharge and follow-up. Though still elevated, her creatinine level was on a decline compared to her first hospitalization. Also, whereas her hemoglobin and albumin levels were still low, they were better at discharge (10.6 g/L and 3.2 g/L respectively) compared to when she was first hospitalized (6.2 g/L and 2.6 g/L respectively).

**Table 2:** Laboratory & Clinical Findings

	Initial hospitalization	Re-hospitalization	At discharge	Follow-up (May, at the age of 52 years)
Height (cm)	149	149	149	149
Weight (kg)	26.4	33.7	45.4	42.0
BMI (kg/m^2^)	11.9	15.2	20.4	18.9
Physical findings	Remarkable emaciation	Marked Edema in both lower thighs	No noteworthy findings	No noteworthy findings
Chest X-ray	CTR 25%	CTR 42.7%	CTR 49.4%	CTR 47.2%
Body temperature (°C)	35.7	36.9	36.4	n.a
Blood pressure	79/49	66/36	107/63	n.a
Pulse rate (times/min adjustment)	92	84	98	n.a
Blood tests [reference values]				
RBC (×10^6^ /μl) [3.86–4.92]	1.97 ↓	2.55 ↓	3.44 ↓	3.39 ↓
Hb (g/dl) [11.6–14.8]	6.2 ↓	8.2 ↓	10.6 ↓	10.1 ↓
TP (g/dl) [6.6–8.1]	4.2 ↓	4.7 ↓	5.7 ↓	6.1 ↓
Alb (g/dl) [4.1–5.1]	2.6 ↓	1.7 ↓	3.2 ↓	3.1 ↓
BUN (mg/dl) [8–20]	10	22 ↑	48 ↑	15
Cre (mg/dl) [0.46–0.79]	5.72 ↑	4.43 ↑	7.25 ↑	3.51 ↑
Na (mEq/l) [138–145]	130 ↓	137 ↓	139	140
K (mEq/l) [3.6–4.8]	2.5 ↓	3.0 ↓	4.1	4.0
Cl (mEq/l) [101–108]	98 ↓	102	105	105
Ca (mg/dl) [8.8–10.1]	8.4 ↓	9.2	8.8	9.2
fT3 (pg/ml) [2.1–3.8]	1.55 ↓	1.49 ↓	n.a.	n.a.
fT4 (ng/ml) [0.82–1.63]	1.06	0.41 ↓	n.a.	1.35
TSH (μIU/ml) [0.38–4.31]	10.02 ↑	2.71	n.a.	11.20 ↑

*n.a* Not available

**Fig. 3:**
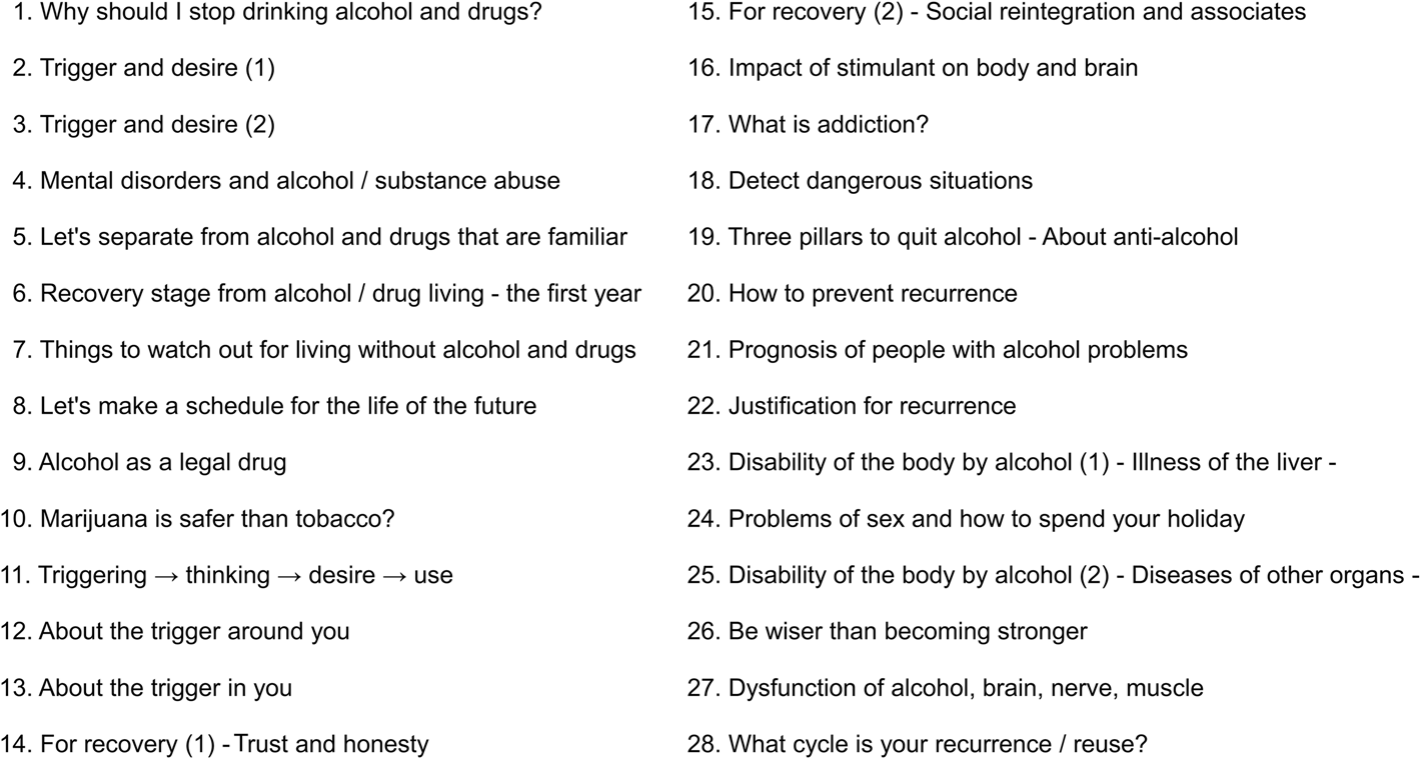
Contents of the SMARPP workbook

**Fig. 4:**
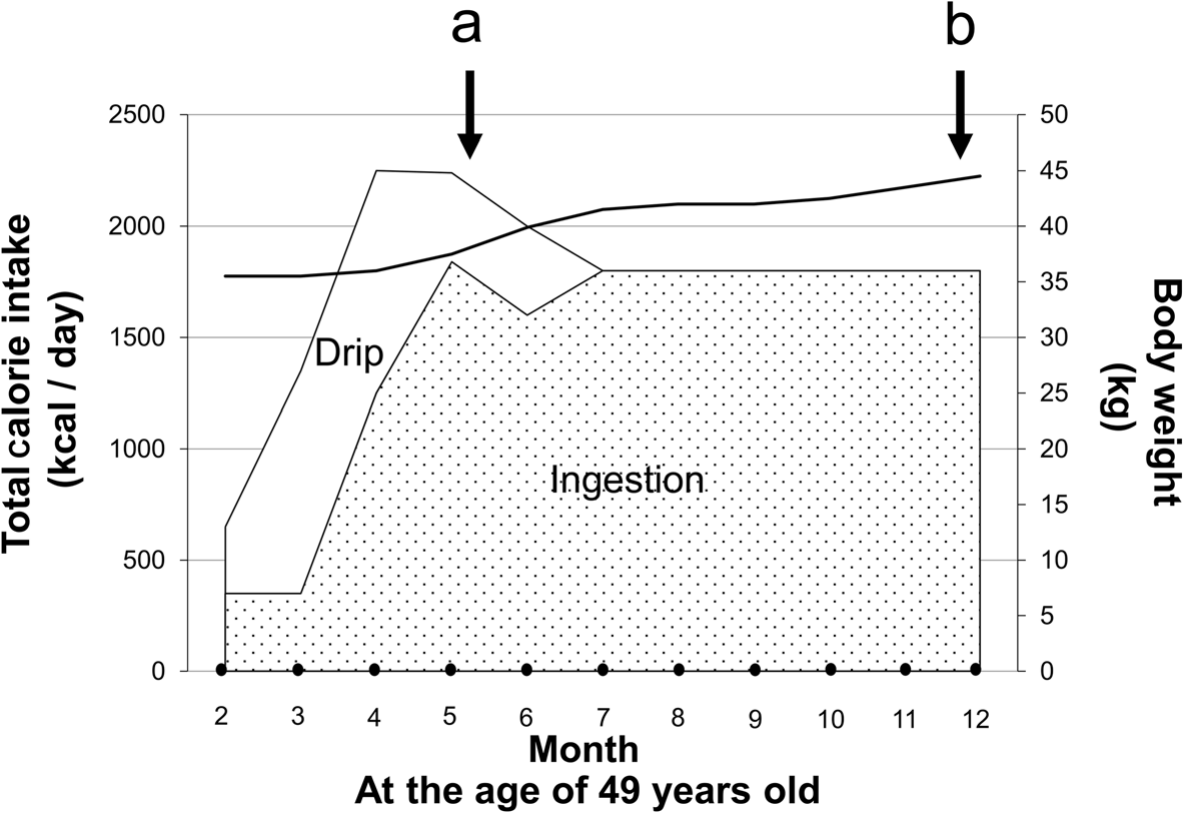
Progress chart following hospitalization. The patient’s progress following the adoption of the SMARPP workbook showed that her weight gain and food intake were stable. Abbreviations: **a**, SMARPP workbook started; **b**, SMARPP workbook finished

Before the SMARPP workbook was adopted, the patient underreported the amount of laxative she used. However, once the workbook was implemented, she provided the correct laxative dose. She also admitted her laxative abuse to medical staff and family members. From that point, she began to work independently to prevent the recurrence of laxative abuse. Figure 3 presents the contents of the SMARPP workbook. In addition, her progress following the adoption of the workbook showed that her weight gain and food intake were stable. This is depicted in Fig. 4.

## Discussion and conclusions

It has been observed that AN with a bingeing component increases a patient’s vulnerability to a substance use disorder [1]. Seen against this background, the present case is significant, as it provides an empirical perspective on laxative abuse in the context of AN-BP. This reinforces the idea that a behavioral and neurobiological correlation exists between AN and addiction [14]. However, it is still a novel concept because the laxative abuse of patients with eating disorders is generally not considered a form of addiction [15, 16].

In this case, we found that the patient’s laxative abuse improved when the SMARPP workbook was used as a therapeutic tool. Because the patient’s serum potassium concentration normalized following the amelioration of laxative abuse, we excluded other differential diagnoses, such as renal tubular acidosis, which may be seen in autoimmune conditions, including Sjögren’s syndrome or hereditary renal tubular disorders [17, 18]. In the treatment of eating disorders, a referral for psychiatric treatment may sometimes help decrease the patient’s reliance on laxatives [15]. Although a correlation exists between laxatives and anxiety in bulimia nervosa (BN) [19], laxative misusers with AN tend to abuse laxatives more than those with BN [20]. However, compared to the number of reports on the effective treatment of laxative abuse for BN, few reports focus on an effective treatment for laxative abuse for those with AN [3].

Due to the effect of the associated low body weight, AN has a lower cognitive component compared to BN [21]. This suggests that a complicated psychological intervention for AN patients with low body weight is difficult. The SMARPP workbook appears quite easy to use and as such may be started from the low weight spectrum of AN. As noted earlier, laxative abuse by older adults may require more external intervention compared to adolescent abusers, who are likely to have others nearby who can provide moral and emotional support; thus, we believe that it may be better to formulate interventions for laxative abuse by middle-aged patients from a drug-dependence perspective.

The combination of self-induced vomiting and laxative–diuretic abuse is more typical in males than in females [22]. The presentation of female AN patients with these symptoms may therefore suggest the need for a thorough clinical history as well as the possibility of employing an addiction intervention strategy, such as SMARPP. However, because this is a case report, this idea cannot be generalized. Nevertheless, the acknowledgment of laxative abuse as a drug addiction in the present case led to the patient’s motivation for treatment, which resulted in an excellent prognosis. Because aberrant cognitions related to eating and shape alter the functioning of central reward systems and reward processing, particularly in the context of illness-compatible cues, these may be exploited to develop treatment and preventive approaches to AN [23] and suggest the clinical value of the SMARPP workbook. This may also explain why the SMARPP workbook has been identified as a convenient tool for increasing patients’ awareness and motivation in the treatment of drug addiction [24]. It remains to be seen whether the recently reported web-based relapse prevention program “e-SMARPP” will exert this same effect [25].

Despite the fact that laxative abuse in AN-BP sufferers is a clinically serious problem, no effective evidence-based treatment has been established. This case report reveals that using the SMARPP workbook for the treatment of drug dependence could be effective in treating laxative abuse in patients with AN-BP (Table 2 and Fig. 4). We assert that if the duration of the illness is long, a dependence on laxatives has been formed or the patient’s cognitive function is declining; hence, the SMARPP workbook may be effective. Nevertheless, future study of similar cases to assess the efficacy of the SMARPP workbook on a larger scale is necessary. Whereas this may be the first report in which the SMARPP workbook is used to treat an eating disorder, and it is imperative to study more cases before generalizable conclusions can be drawn for its use in patients with AN-BP.

In this case, the evidence of recovery from eating disorder pathology was limited because SCOFF is usually used for screening of eating disorders. However, the psychopathology of eating disorder cannot be measured by this tool. In future studies, further objective assessment should be performed to confirm whether the SMARPP workbook contributes to the improvement of eating disorder pathology.

## Data Availability

Data sharing is not applicable to this article as no datasets were generated or analyzed during the current study.

## Abbreviations

AN: Anorexia nervosa
AN-BP: Anorexia nervosa-binge eating/purging type
BMI: Body mass index
BN: Bulimia nervosa
SMARPP: Serigaya Methamphetamine Relapse Prevention Program
